# Dual and Triple Epithelial Coculture Model Systems with Donor-Derived Microbiota and THP-1 Macrophages To Mimic Host-Microbe Interactions in the Human Sinonasal Cavities

**DOI:** 10.1128/mSphere.00916-19

**Published:** 2020-01-15

**Authors:** Charlotte De Rudder, Marta Calatayud Arroyo, Sarah Lebeer, Tom Van de Wiele

**Affiliations:** a Center for Microbial Ecology and Technology, Faculty of Bioscience Engineering, Ghent University, Ghent, Belgium; b Research Group of Environmental Ecology and Applied Microbiology, Department of Bioscience Engineering, University of Antwerp, Antwerp, Belgium; University of Iowa

**Keywords:** host-microbe interaction, microbiome, respiratory epithelium, upper respiratory tract

## Abstract

The epithelium of the human sinonasal cavities is colonized by a diverse microbial community, modulating epithelial development and immune priming and playing a role in respiratory disease. Here, we present a novel *in vitro* approach enabling a 3-day coculture of differentiated Calu-3 respiratory epithelial cells with a donor-derived bacterial community, a commensal species (Lactobacillus sakei), or a pathobiont (Staphylococcus aureus). We also assessed how the incorporation of macrophage-like cells could have a steering effect on both epithelial cells and the microbial community. Inoculation of donor-derived microbiota in our experimental setup did not pose cytotoxic stress on the epithelial cell layers, as demonstrated by unaltered cytokine and lactate dehydrogenase release compared to a sterile control. Epithelial integrity of the differentiated Calu-3 cells was maintained as well, with no differences in transepithelial electrical resistance observed between coculture with donor-derived microbiota and a sterile control. Transition of nasal microbiota from *in vivo* to *in vitro* conditions maintained phylogenetic richness, and yet a decrease in phylogenetic and phenotypic diversity was noted. Additional inclusion and coculture of THP-1-derived macrophages did not alter phylogenetic diversity, and yet donor-independent shifts toward higher *Moraxella* and *Mycoplasma* abundance were observed, while phenotypic diversity was also increased. Our results demonstrate that coculture of differentiated airway epithelial cells with a healthy donor-derived nasal community is a viable strategy to mimic host-microbe interactions in the human upper respiratory tract. Importantly, including an immune component allowed us to study host-microbe interactions in the upper respiratory tract more in depth.

**IMPORTANCE** Despite the relevance of the resident microbiota in sinonasal health and disease and the need for cross talk between immune and epithelial cells in the upper respiratory tract, these parameters have not been combined in a single *in vitro* model system. We have developed a coculture system of differentiated respiratory epithelium and natural nasal microbiota and incorporated an immune component. As indicated by absence of cytotoxicity and stable cytokine profiles and epithelial integrity, nasal microbiota from human origin appeared to be well tolerated by host cells, while microbial community composition remained representative for that of the human (sino)nasal cavity. Importantly, the introduction of macrophage-like cells enabled us to obtain a differential readout from the epithelial cells dependent on the donor microbial background to which the cells were exposed. We conclude that both model systems offer the means to investigate host-microbe interactions in the upper respiratory tract in a more representative way.

## Abstract (precis)

Despite the relevance of the resident microbiota in sinonasal health and disease and the need for cross talk between immune and epithelial cells in the upper respiratory tract, these parameters have not been combined in a single *in vitro* model system. We have developed a coculture system of differentiated respiratory epithelium and natural nasal microbiota and incorporated an immune component. As indicated by absence of cytotoxicity and stable cytokine profiles and epithelial integrity, nasal microbiota from human origin appeared to be well tolerated by host cells, while microbial community composition remained representative for that of the human (sino)nasal cavity. Importantly, the introduction of macrophage-like cells enabled us to obtain a differential readout from the epithelial cells dependent on the donor microbial background to which the cells were exposed. We conclude that both model systems offer the means to investigate host-microbe interactions in the upper respiratory tract in a more representative way.

## INTRODUCTION

In constant exposure to the external environment, the upper airways, and more specifically the sinonasal cavities, are body sites with intense host-microbe interaction and substantial allergen exposure. It is therefore unsurprising that upper airway diseases are one of the main reasons for family physician visits (1). To advance research on respiratory disease several *in vitro* and *in vivo* model systems have been created (reviewed in O’Leary et al. [2] and Shin [3]). *In vitro* human cell-based assays are routinely used to examine disease etiopathology and explore the potential of new preventive and therapeutic applications for abating (chronic) airway diseases.

Differentiated air-liquid interface (ALI) cultured airway epithelium is widely used to mimic *in vivo* airway epithelial structures in the lab. It has been validated for the presence of relevant structural and functional physiological parameters such as cilium formation, epithelial barrier integrity, the presence of tight and adherence junctions, cytokine secretion, mucus production, and mucociliary clearance (4–8). ALI cultured Calu-3 cells, a bronchial epithelial cell line, display more features of the *in vivo* epithelium than submerged cultured Calu-3 cells. Apical exposure to air results in more and longer cilia, increased mucus production, and the pseudostratified structure observed in upper respiratory tract (URT) epithelium (7, 9). Stewart et al. (10) found that ALI cultured Calu-3 cells had a pattern of epithelial markers better resembling ALI cultured primary bronchial cells than other bronchial epithelial cell lines and were the only ones tested able to develop representative high epithelial resistance (≥500 Ω). ALI culture is also important for primary cells from tracheal and bronchial origin; these display a transcriptional profile that is more similar to *in vivo* epithelium than submerged cultured primary cells (11). The differentiation of lab-grown cells is as important as the type of cells. This was demonstrated by a previous report where exposure to flagellin triggered differentiated cell layers to a transcriptional response that is physiologically more relevant for healthy airway epithelium than nondifferentiated monolayers, which behaved similar to epithelium undergoing repair (12). These findings underline the necessity of an appropriate cell culture method to study host-microbe interactions.

Another important parameter to consider for inclusion in upper respiratory tract model systems is the endogenous microbiota. Microbial populations in and on the sinonasal cavity and epithelium play an important role in immune priming and epithelial development (reviewed in Belkaid and Hand [13] and Salzano et al. [14]) and have been proposed to play a modulating role in chronic airway diseases such as chronic rhinosinusitis (15, 16). Jain et al. (17) discovered functional and structural differences in the paranasal sinus epithelium between germfree and specific-pathogen-free mice, suggesting the importance of a healthy microbiota in normal epithelial development. In addition, the sinonasal microbial community can act as a colonization barrier to incoming microorganisms or prevent overgrowth of pathogenic species (18, 19). Pathogenic species (16, 20) encounter a diverse polymicrobial community present on the epithelium, which may facilitate or block their adherence and outgrowth. Similarly, nasally administered probiotics (21, 22) will have to be introduced in a polymicrobial environment where they have to be able to exert their beneficial mode of action. Most current upper respiratory tract model systems do not take into account such polymicrobial background and limit the number of microbial species that are in interaction with the host cells (23–27). We recently emphasized that a physiologically relevant model system of the sinonasal microenvironment requires both a representative epithelial structure and a microbial community that is characteristic for that of the human nose and sinuses (28). Charles et al. (29) have incorporated these parameters in their model system and have developed an *ex vivo* nasal model that supports colonization of nasal bacteria on a cultured host mucosa created by immortalized human nasal epithelial cells.

Macrophages might have a reinforcing role in disease development in upper airway diseases such as chronic rhinosinusitis with nasal polyps (CRSwNP). Larger amounts of (alternatively activated) macrophages were found in CRS polyps compared to control tissue (30–33), as well as in eosinophilic CRSwNP polyps compared to noneosinophilic polyps and control tissue (34). As demonstrated by Schulz et al. (35), intercellular cross talk between airway epithelial cells and macrophages is important in modulating the inflammatory response. These researchers showed that preinfected THP-1-derived macrophage-like cells have immunomodulatory capacities toward the response of airway epithelial cells during Legionella pneumophila infection. It was proposed that this is a mechanism to avoid excessive inflammation. Next to this, pretreatment with probiotic bacteria (Lactobacillus rhamnosus LR32, Lactobacillus acidophilus NCFM, and potential probiotic Lactobacillus casei L324m) could modulate the response of macrophage-like cells toward Candida albicans infection (36). Both studies emphasize the importance of considering intercellular communication between the epithelium, immune cells, and microorganisms in research on airway diseases.

With the resident microbiota and the need for intercellular cross talk, we have identified two essential parameters in host-microbe research in the URT that, despite their relevance in the etiopathology of certain URT diseases, have to the best of our knowledge not been combined in a single model system. In the present study, we developed and tested two coculture model systems to investigate host-microbe interactions in the human upper respiratory tract. In a first step, we developed a dual coculture model system based on differentiated airway epithelial cells (Calu-3) at ALI and apically exposed to a natural nasal community, Staphylococcus aureus, a CRS-relevant pathogen (37, 38), Lactobacillus sakei, a commensal or potentially probiotic species (20), or a sham-inoculated control. In a next step, we expanded our model system with THP-1-derived macrophages, the triple coculture setup. We evaluated what impact the presence of this immune component has on the epithelial cells and on microbial populations from human donors.

## RESULTS

### Dual coculture of epithelial cells with nasal microbiota. (i) Cytotoxicity.

**FIG 1:**
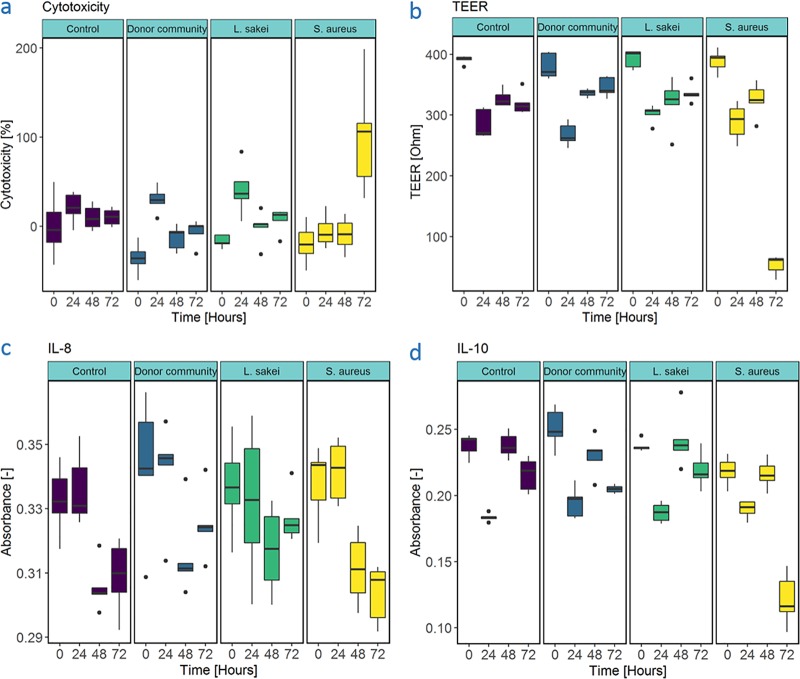
Host responses during dual coculture. (a) Percent cytotoxicity during dual coculture. (b) Transepithelial electrical resistance during dual coculture experiment. Time is displayed in hours. TEER is corrected for an empty insert is displayed in Ohm. (c) IL-8 release during dual coculture. IL-8 release is displayed as absorbance at 450 nm, and duration of coculture is presented in hours. (d) IL-10 release during dual coculture. IL-10 release is displayed as absorbance at 405 nm, and duration of coculture is presented in hours.

Cytotoxic stress of the Calu-3 cells exposed to bacterial cultures was determined through lactate dehydrogenase (LDH) release in the basolateral medium. Cytotoxicity remained stable for the control group (no bacteria) during the entire incubation period. After 24 h, the cytotoxicity was mildly increased for the donor- and the *L. sakei*-inoculated Calu-3 cells compared to the start of the experiment, whereas this was not the case for the S. aureus exposed cells after 24 h. A significant difference (as determined by the Kruskal-Wallis test [K-W]) was observed between *L. sakei*-treated cell layers and S. aureus-treated cell layers after 24 h, as well as a (nonsignificant) difference between the cell layers inoculated with donor-derived microbiota and S. aureus (*P* = 0.087), with the S. aureus-treated group displaying lower cytotoxicity in both cases (Fig. 1a). However, after 72 h of coculture with *L. sakei* or nasal microbiota, Calu-3 cells did not display significantly increased cytotoxic stress as opposed to control (no bacteria) conditions. In contrast, a sharp increase in cytotoxicity was noticed for the S. aureus-treated Calu-3 cells. For S. aureus-exposed Calu-3 cells, a significant increase in cytotoxicity was detected. Due to a high standard deviation (SD) at 72 h, readout levels were not significantly higher than the *L. sakei*-treated group (*P* = 0.077) and the control group (*P* = 0.0947).

### (ii) Transepithelial electrical resistance.

Epithelial barrier functioning was assessed by means of transepithelial electrical resistance (TEER) measurement during the differentiation period of 3 weeks (21 days) at air-liquid interface. TEER values, measured with growth medium on both sides, were between 534 ± 2 Ω (average ± SD, *n* = 2) and 744 ± 7 Ω (*n* = 2) at the end of the differentiation period. During the coculture assay, epithelial resistance was examined with phosphate-buffered saline (PBS) instead of minimal essential medium (MEM) in the upper compartment, resulting in lower readouts (between 360 ± 1 Ω and 411 ± 1 Ω) compared to TEER measured with growth medium in both compartments. During the first 48 h of coculture, a similar trend in epithelial resistance could be observed for all wells (Fig. 1b). TEER values were significantly lower for all groups after 24 h compared to 0 h. In general, no significant differences in epithelial resistance were observed between control conditions and the different treatments at 24 or 48 h. However, S. aureus-inoculated cell layers displayed significantly lower epithelial integrity after 72 h compared to the start of the experiment (K-W). This effect was also apparent from the significantly lower TEER value for S. aureus-challenged epithelial cells (52 ± 20 Ω), as opposed to the sterile control condition (319 ± 23 Ω) after 72 h. No such decrease in TEER after 72 h was observed for Calu-3 cells cocultured with nasal microbiota (345 ± 20 Ω) or *L. sakei* (336 ± 19 Ω).

### (iii) Cytokine secretion.

With respect to cytokine secretion, we found the coculture experimental setup, irrespective of its treatment conditions, to display lower interleukin-8 (IL-8) secretion levels after 48 and 72 h compared to the 0- and 24-h values. No differences between treatments (analysis of variance [ANOVA], *P* = 0.0713) were observed (Fig. 1c). In terms of anti-inflammatory IL-10, secretion from cell layers inoculated with nasal microbiota or *L. sakei* did not differ from control conditions (Fig. 1d). However, this was not the case for S. aureus-inoculated cell layers, for which decreasing levels of IL-10 were noted over time. Moreover, IL-10 secretion from the S. aureus-treated Calu-3 cells were significantly lower (*P* < 0.05) than those from the control, nasal microbiota, or *L. sakei* groups (Table 1).

**TABLE 1:** Percentage of IL-10 secretion during dual coculture versus control cell layers at *t* = 0 h

Group	Mean % IL-10 secretion ± SD		
	IL-10 at 24 h	IL-10 at 48 h	IL-10 at 72 h
Control	77 ± 1	100 ± 4	91 ± 5
*S. aureus*	80 ± 3	91 ± 4	51 ± 8
*L. sakei*	79 ± 3	102 ± 9	92 ± 6
Nasal community	82 ± 5	97 ± 6	86 ± 1

### (iv) Bacterial growth and phenotypic diversity.

**FIG 2:**
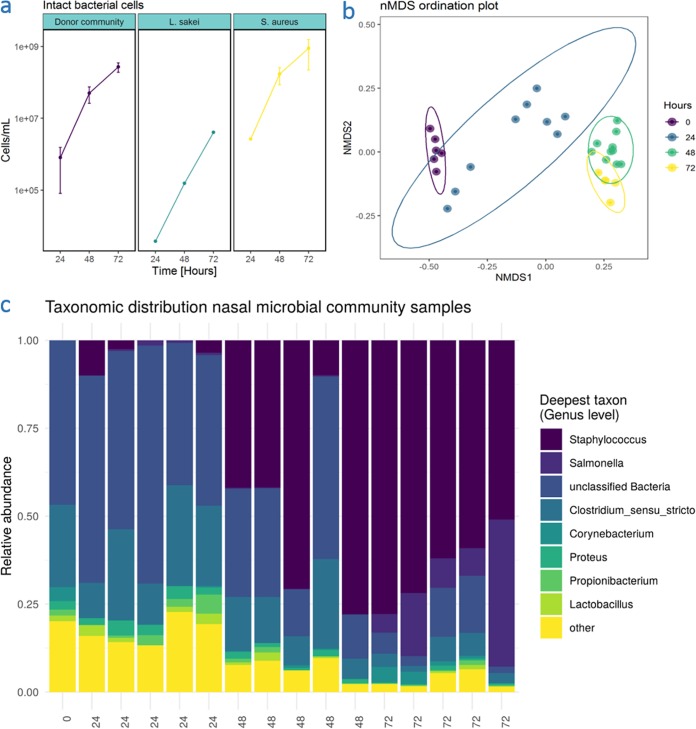
Microbial behavior during dual coculture. (a) Intact bacterial cells in apical washing liquid during proof-of-concept experiment. Intact bacterial cell numbers were determined by flow cytometry with intact/damaged staining. Background caused by cell debris was subtracted from total bacterial counts based on sterile control cell layers. Cell layers were washed using 1 ml of phosphate-buffered solution. (b) NMDS ordination plot based on Bray-Curtis distances between flow cytometric fingerprints of donor-derived communities in apical washes during proof of concept coculture experiment. Time is indicated in hours. Stress = 0.039. (c) Taxonomic distribution of donor-derived nasal bacterial community during proof-of-concept experiment. The *x* axis indicates time in hours, and the *y* axis indicates the relative abundance on the genus level.

Throughout the entire incubation, comparable (*P* = 0.94) bacterial cell densities were measured in the apical washing fluids from the nasal microbiota and S. aureus-treated cell cultures (Fig. 2a). However, bacterial cell density in apical washing fluid from *L. sakei*-treated samples stayed at least 2 orders of magnitude lower than the latter groups (*P* = 0.00095).

The phenotypic alpha diversity of the donor-derived bacterial community (D2, Gini-Simpson index) is presented in Fig. S1 in the supplemental material. The average phenotypic alpha diversity decreased from 3,180 ± 134 (mean ± SD) at the start of the experiment to 1,910 ± 401 after 24 h and further decreased to 950 ± 132 after 48 h to remain at 1,004 ± 86 at 72 h. The phenotypic alpha diversity of the bacterial community was not significantly different from the inoculum after 24 h of coculture; however, this was the case after 48 and 72 h of coculture (*P* < 0.001, K-W).

10.1128/mSphere.00916-19.1 FIG S1 Phenotypic alpha diversity (D2) of donor-derived bacterial community during dual coculture. Time is displayed in hours. Download FIG S1, TIF file, 0.9 MB. Copyright © 2020 De Rudder et al. 2020 De Rudder et al. This content is distributed under the terms of the Creative Commons Attribution 4.0 International license.

These findings are indicative of an ecological shift during the coculture experiment: this was also reflected by the time-dependent clustering of the microbial population as based on phenotypic beta-diversity (nonmetric multidimensional scaling [NMDS], stress = 0.039; Fig. 2b). Although microbial communities 24 h postinoculation remained more similar to the inoculum, communities at 48 and 72 h clearly differed from those at 0 and 24 h.

### (v) Microbial community analysis.

Partial 16S rRNA genes were sequenced from swab and coculture samples, taken every 24 h. Relative abundance of bacterial genera is displayed in Fig. 2c. Richness was estimated as Chao1 (39) for all samples. Estimated richness of the swab sample (*t* = 0 h) was 35. The average estimated richness values (± SD) at 24, 48, and 72 h were 44.6 ± 19.5, 42.6 ± 6.4, and 52.8 ± 16.6, respectively. The duration of coculture did not significantly affect Chao1 richness (*P* > 0.05, ANOVA). Alpha diversity was estimated using the Shannon index and the inverse Simpson index (Table 2). A significant effect of duration of coculture on Shannon diversity was observed (*P* = 0.02718). Bacterial communities at *t* = 72 h had a lower Shannon diversity than bacterial communities at 24 h (Tukey’s honestly significant difference [HSD], *P* = 0.022, estimate = −0.76). Differences in inverse Simpson diversity were not significant (*P* = 0.07951, K-W), notwithstanding a trend toward lower inverse Simpson indices at later time points.

**TABLE 2:** Estimated richness (Chao1) and diversity (Shannon index and inverse Simpson index) of the bacterial community during dual coculture

Time (h)	Avg ± SD		
	Chao1	Shannon index	Inverse Simpson index
0	35.0	1.7	3.5
24	44.6 ± 19.5	2.0 ± 0.3	3.7 ± 0.7
48	42.6 ± 6.4	1.4 ± 0.4	2.6 ± 0.8
72	52.8 ± 16.6	1.2 ± 0.3	2.1 ± 0.4

There was a significant effect of the duration of coculture on beta diversity, whereas there was no significant difference between biological replicates (Fig. S2). No significantly different operational taxonomic units (OTU) were observed between the bacterial community at inoculation (*t* = 0 h) and after 24 h of coculture. Bacterial communities at *t* = 48 h and *t* = 72 h displayed increases in Otu0001 (48 h) and in Otu0001, Otu0002, and Otu0005 (72 h) compared to the inoculum. Otu0002 was increased in bacterial communities after 72 h compared to bacterial communities after 48 h of coculture. Otu0001 was classified as Staphylococcus epidermidis (BLAST), *S. caprae* (SeqMatch), or S. epidermidis*/*S. capitis (eHOMD). Otu0002 was classified as Salmonella enterica by BLAST and SeqMatch and as Klebsiella pneumoniae by eHOMD. Otu0005 was classified as *Mycoplasma* sp. (BLAST), *M. weynonii* (SeqMatch), and M. genitalium (eHOMD).

10.1128/mSphere.00916-19.2 FIG S2 NMDS plot constructed using Bray-Curtis distances of community composition of apical wash samples during dual coculture (stress = 0.0377). Clustering of samples is based on time (in hours). Labels: t0 = 0 h, t3 = 24 h, t5 = 48 h, t7 = 72 h. Download FIG S2, TIF file, 0.04 MB. Copyright © 2020 De Rudder et al. 2020 De Rudder et al. This content is distributed under the terms of the Creative Commons Attribution 4.0 International license.

### Triple coculture of respiratory epithelial cells, immune cells, and nasal microbiota. (i) Cytotoxicity.

**FIG 3:**
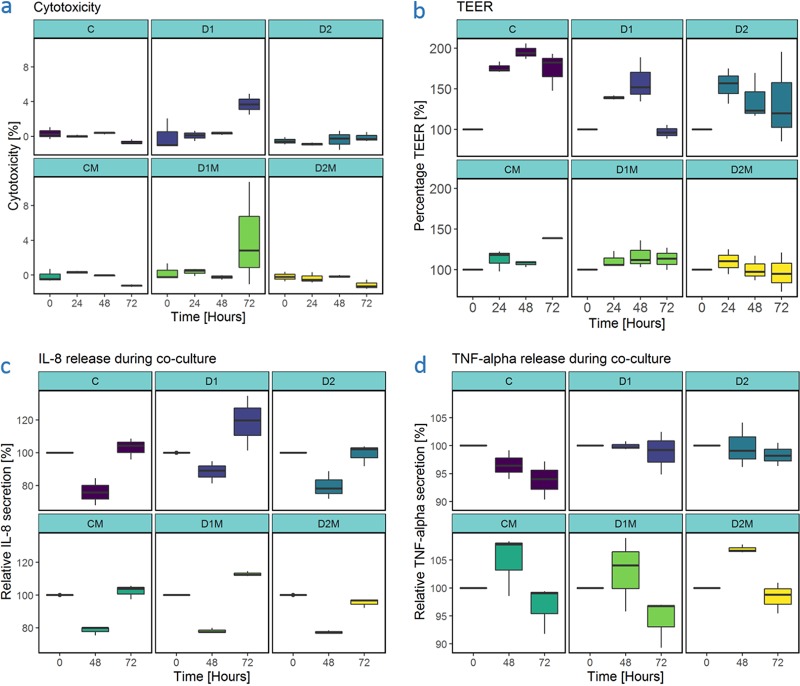
Host responses during triple coculture experiment. (a) Cytotoxicity during triple coculture experiment. Duration of coculture is presented in hours. (b) Transepithelial electrical resistance during triple coculture experiment. TEER is expressed in percentage of the respective cell layers at *t* = 0 h. Time is displayed in hours. (c) IL-8 release during triple coculture study. IL-8 release is displayed as the percentage of the concentration at *t* = 0 h. (d) TNF-α release is displayed as the percentage of the concentration at *t* = 0 h. Abbreviations: C, control; D, donor-inoculated cell layers, with “1” indicating donor 1 and “2” indicating donor 2; M, coculture with THP-1-derived macrophage-like cells.

Cytotoxicity levels, as measured by LDH release, were low (on average below 10%) during the 72 h of incubation. We observed no significant differences in cytotoxicity between Calu-3 cell layers in dual or triple cocultures inoculated with material from the same donor; hence, the addition of macrophages to the setup did not modulate cytotoxicity levels. Cytotoxicity did not differ significantly between different inocula for Calu-3 cell layers in dual cocultures, nor was this the case for Calu-3 cell layers in triple cocultures (Fig. 3a). However, cell layers inoculated with nasal microbiota from donor 1 displayed higher cytotoxicity than cell layers with material from donor 2 at 72 h, in both dual and triple cocultures.

In an additional experiment, triple coculture experiments were performed using L. sakei or S. aureus as the inoculum, with a sham-inoculated (sterile PBS) cell layer as the control. There were no differences in cytotoxicity (see Fig. S3a in the supplemental material) at the start of the experiment, whereas at 24 h, the *L. sakei* group had a significantly higher, albeit low, cytotoxicity percentage (1.31 ± 0.96%) than the control group (−0.79 ± 0.50%). After 72 h of coculture, S. aureus-inoculated cell layers displayed a significantly (K-W) higher cytotoxicity percentage (42.10 ± 10.31%) than the control group (−1.48 ± 0.36%). *L. sakei*-inoculated cell layers retained low cytotoxicity percentages comparable to the control at this time point (2.21 ± 0.63%).

10.1128/mSphere.00916-19.3 FIG S3 Host responses during triple coculture with S. aureus, *L. sakei*, or a sterile control. (a) Cytotoxicity based on lactate dehydrogenase release. (b) Transepithelial electrical resistance (TEER), calculated as the percentage of TEER of respective cell layers at *t* = 0 h. (c) TNF-α secretion during coculture. Samples indicated by a double dagger (‡) were below the limit of detection. (d) IL-8 secretion during coculture. Samples indicated by an asterisk (*) were above the quantification limit of the spectrophotometer and could not be quantified. Download FIG S3, TIF file, 0.1 MB. Copyright © 2020 De Rudder et al. 2020 De Rudder et al. This content is distributed under the terms of the Creative Commons Attribution 4.0 International license.

### (ii) Transepithelial electrical resistance.

During the differentiation period at ALI, TEER values were measured every fourth or fifth day. The average TEER value was 515 ± 71 Ω (average ± SD) at the end of the differentiation period. During the coculture assay, epithelial resistance was examined with PBS instead of MEM in the upper compartment, resulting in lower readouts compared to TEER measured with growth medium in both compartments. At the start of the experiment, the average TEER value measured in this manner was 283 ± 81 Ω. The evolution of epithelial resistance is presented in Fig. 3b (percentages) and in Fig. S4. At the start of the experiment, all cell layers were transferred from MEM to the richer RPMI medium to allow coculture with THP-1 cells. This resulted in an increase in epithelial resistance from *t* = 0 h to *t* = 24 h. Differences between time points per treatment group were not significant (*P* > 0.05, K-W). No significant differences in TEER between Calu-3 cell layers in dual and triple cocultures were observed for the different donor groups (*P* > 0.05, K-W), although a noticeable trend exists for lower TEER percentages in Calu-3 cell layers in triple coculture.

10.1128/mSphere.00916-19.4 FIG S4 Transepithelial electrical resistance during triple coculture experiment. Time is displayed in hours. TEER is expressed in ohms. Abbreviations: C, control; D, donor-inoculated cell layers, with “1” indicating donor 1 and “2” indicating donor 2; M, coculture with THP-1-derived macrophage-like cells. Download FIG S4, TIF file, 0.01 MB. Copyright © 2020 De Rudder et al. 2020 De Rudder et al. This content is distributed under the terms of the Creative Commons Attribution 4.0 International license.

In an additional experiment, triple coculture experiments were performed using *L. sakei* or S. aureus as inoculum, with sham-inoculated (sterile PBS) cell layers as control group. We found no significant differences in TEER values or TEER percentages (Fig. S3b) between the *L. sakei*-inoculated group and the control group, whereas TEER values of S. aureus-inoculated cell layers were significantly lower than those of the control group after 48 h of coculture (K-W). This difference was noticeable but not significant (*P* = 0.09, *post hoc* after K-W) after 72 h of coculture.

### (iii) Cytokine secretion.

The presence of macrophage-like cells has a minor impact on IL-8 release over time (*P* = 0.43). However, a significant interaction effect between the origin of the inoculum and the presence of macrophage-like cells was observed [F(2,1)= 13.70, *P* < 0.0001] (Fig. 3c). The inoculum (donor 1, donor 2, or control) had a significant effect on IL-8 release (*P* < 0.0001). The impact of macrophage-like cells is dependent on the donor background. In donor 1, the introduction of macrophage-like cells results in a sharp increase in IL-8 (*P* = 0.0001), while for donor 2 more stable IL-8 levels are observed, with a mild decrease at 72 h. The presence of THP-1-derived macrophages had a significant impact on tumor necrosis factor alpha (TNF-α) release (Fig. 3d) for each donor (K-W), with increased TNF-α production in triple coculture setups. There was no overall difference in TNF-α release for the sterile control groups between dual and triple cocultures, indicating that TNF-α release was induced by exposure to the nasal microbiota. TNF-α reached a peak after 48 h of coculture. IL-10 release was below the detection limit (23 pg/ml) for all samples (data not shown).

In an additional experiment, triple coculture experiments were performed using *L. sakei*, S. aureus, or sterile PBS as a (sham) inoculum. We observed no differences (K-W) in TNF-α production at the start of the experiment, whereas after 48 and 72 h of coculture, TNF-α secretion differed significantly between the treatments (Fig. S3c). TNF-α secretion in S. aureus-treated cell layers was severely increased in comparison to *L. sakei-*treated and control cells layers after 48 and 72 h of coculture. Although IL-8 release did not differ between groups at the start of the assay, S. aureus-treated cell layers reached IL-8 secretion well over 3,500 pg/ml from 24 h of coculture on. Cell layers cocultured with *L. sakei* had a significantly higher IL-8 release than the sterile control at 24 h after the start of the experiment, but after 48 and 72 h of coculture the IL-8 release was comparable to that of the sterile control group (Fig. S3d). IL-10 secretion was below the quantification limit for all samples (data not shown).

### (iv) Bacterial growth and phenotypic diversity.

**FIG 4:**
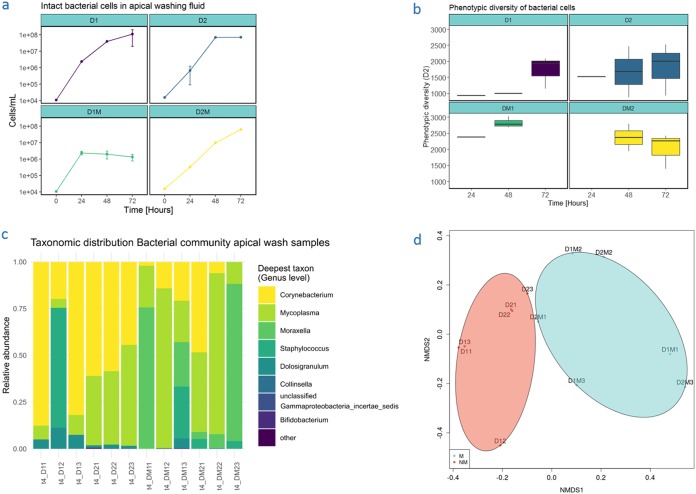
Microbial behavior during triple coculture. (a) Intact bacterial cells in apical washing fluid during triple coculture experiment. Time is indicated in hours. D, donor inoculated cell layers, with “1” indicating donor 1 and “2” indicating donor 2. M, coculture with THP-1-derived macrophage-like cells. (b) Phenotypic alpha diversity of bacterial cells in apical washing fluid. Samples with insufficient data points to calculate phenotypic diversity were omitted. Time is indicated in hours. (c) Taxonomic distribution of donor-derived bacterial communities after 72 h of coculture, sampled through apical washes during the triple coculture experiment. The relative abundance on the genus level is displayed on the *y* axis. Sample names are displayed on the *x* axis, with M indicating the presence of macrophage-like cells, the first number indicating donor 1 or donor 2, and the last number indicating the replicate. (d) NMDS ordination plot constructed using Bray-Curtis distances of sample composition (stress = 0.063). A clustering based on presence (M) or absence (NM) of macrophage-like cells can be observed. Sample names are indicated, with M indicating the presence of macrophage-like cells, the first number indicating donor 1 or donor 2, and the last number indicating the replicate. Colors: teal, triple coculture communities; coral, dual coculture communities.

Bacterial cell density in the apical washing fluid (Fig. 4a) did not significantly differ between donors or dual or triple coculture systems. However, a clear stagnation in bacterial cell numbers can be observed in the triple coculture system for donor 1, whereas this is not the case in the dual coculture for this donor.

In an additional experiment, triple coculture experiments were performed using *L. sakei*, S. aureus, or sterile PBS as (sham) inoculum. Intact cell densities of S. aureus in the apical washing liquid increased to 1.48 × 10^8^ cells ml^−1^ ± 1.53 × 10^7^ cells ml^−1^ 24 h after inoculation and further increased to 5.05 × 10^8^ cells ml^−1^ ± 3.06 × 10^8^ cells ml^−1^ after 72 h of coculture. *L. sakei* intact cell counts were 1.08 × 10^6^ cells ml^−1^ ± 4.68 × 10^5^ cells ml^−1^ 24 h after inoculation and showed a mild increase to 1.60 × 10^6^ cells ml^−1^ ± 5.72 × 10^5^ cells ml^−1^ after 72 h of coculture, which is ca. 2 orders of magnitude lower than the pathobiont. These cell counts are similar to those observed in dual cocultures.

The phenotypic alpha diversity (D2, Gini-Simpson index) is presented in Fig. 4b. The average phenotypic alpha diversities over all time points for donor 1 on Calu-3 without macrophage-like cells were 1,422 ± 553 (average ± SD) and 2,716 ± 268 with macrophage-like cells. For donor 2, average phenotypic alpha diversities on Calu-3 without macrophage-like cells were 1,735 ± 734 and 2,191 ± 478 with macrophage-like cells. The origin of the inoculum did not have a significant effect on the alpha diversity, nor was the interaction term significant. The presence of macrophage-like cells had a significant effect on the phenotypic alpha diversity (*P* = 0.003). Microbial communities present on Calu-3 cell layers cocultured with THP-1 cells had a higher phenotypic alpha diversity than did microbial communities present on Calu-3 cell layers without THP-1 cells (Tukey HSD, *P* = 0.004; estimate, 808.8).

No clustering of samples by similarity of phenotypic beta-diversity could be observed based on the origin of the inoculum (donor) or the presence or absence of macrophage-like cells (NMDS ordination, stress = 0.19).

### (v) Microbial community analysis.

DNA was extracted from samples taken after 72 h of coculture. Samples at 0, 24, and 48 h did not contain enough DNA for sequencing. Richness was estimated as Chao1 [47] for all samples (Table 3). The relative abundance of bacterial genera in the samples is displayed in Fig. 4c. The alpha diversity was estimated using the Shannon index and the inverse Simpson index (Table 3). We observed no significant differences in inverse Simpson index and Shannon index between communities from the same donor with or without macrophage-like cells or between identical setups with different inocula (*P* > 0.5, K-W).

**TABLE 3:** Estimated richness (Chao1) and diversity (Shannon index and inverse Simpson index) of the bacterial community during triple coculture

Donor	Presence or absence of macrophages	Avg ± SD^*a*^		
		Chao1	Shannon index	Inverse Simpson index
1	Absent	36.0 ± NA	0.69 ± 0.28	1.63 ± 0.45
	Present	25.0 ± 10	0.87 ± 0.58	2.44 ± 1.68
2	Absent	5.0 ± NA	0.77 ± 0.01	2.01 ± 0.04
	Present	42.0 ± 31.1	0.68 ± 0.27	1.70 ± 0.59

a NA, not applicable.

There was a significant effect of presence or absence of macrophage-like cells (*P* < 0.05, Fig. 4d) on the beta diversity, whereas the origin of the inoculum (donor 1 or 2) did not significantly affect clustering, nor was the interaction between both significant.

Differences in OTU abundance between samples obtained from cocultures with or without macrophage-like cells and between samples from different donors were assessed using differential expression analysis by means of the DESeq2 package (40) and were found to be not significant (*P* > 0.5). However, a clear trend can be observed in the composition of communities on genus level in dual versus triple coculture setups. Bacterial communities in dual coculture were dominated by *Corynebacterium* species, whereas communities retrieved from a triple culture setup were characterized by high abundances of *Moraxella* and *Mycoplasma* species.

## DISCUSSION

To advance our understanding of host-microbe interactions in the upper respiratory tract (URT) diseases, we have developed an *in vitro* coculture model that incorporates respiratory epithelial cells with two modulatory components of URT disease etiology: microbiota and macrophages.

With respect to the microbiota as modulatory component, we succeeded in maintaining Calu-3/microbiota cocultures for 72 h. It is important to note that we exposed the Calu-3 cells to microbiota at *in vivo* representative concentrations of 10^3^ bacterial cells (41) per well, whether in pure culture or as complex microbiota from nasal swabs. Together with the mucus present on the differentiated Calu-3 cells, this limits the profound and immediate cytotoxicity that is typically observed when exposing cells to high levels of microbiota. For example, wild-type S. aureus at 10^8^ cells ml^−1^ reduced epithelial integrity in direct-contact cocultures with differentiated Calu-3 cell layers within 4 h (25), and treatment with conditioned medium from this bacterium resulted in 90 to 100% cytotoxicity in differentiated Calu-3 cells after 24 h. This is in sharp contrast with our coculture assay, where Calu-3 cells were in contact with bacteria and yet remained viable for more than 48 h. In the coculture setup of Charles et al. (29), consisting of differentiated primary nasal epithelial cell layers apically inoculated with 5 × 10^2^ methicillin-resistant S. aureus organisms, unconstrained growth of this bacterium was observed after 48 h, and yet the authors of that study observed that the colonization appeared to remain limited to the apical surface, without cell layer invasion. This is consistent with our observation that severe damage to epithelial cell layers cocultured with S. aureus was not observed after 48 h. Calu-3 cells exposed for 72 h with S. aureus displayed cytotoxic stress, concomitantly with a decrease in epithelial integrity and lower production of anti-inflammatory cytokine IL-10. Similar S. aureus cell concentrations were observed in apical washing fluids as the natural nasal community (where no cytotoxic stress or drop in TEER was seen at 72 h). We therefore assume that the observed cytotoxic response is independent of bacterial load but is rather caused by S. aureus’ pathogenicity. In the absence of an extracellular matrix (42, 43) or growth-promoting signaling from other cell types (fibroblasts or macrophages) (42, 44), repair of the epithelial layer during pathogen colonization was not sufficiently supported. This underlines the limitations of monoculture model systems for prolonged host-microbe interaction, in particular for pathogenic microorganisms.

In contrast to S. aureus, 72 h of incubation of Calu-3 cells with *L. sakei* or a healthy donor-derived microbial community did not result in cytotoxicity, nor did it decrease epithelial barrier function. The microbiota from the nasal swabs from healthy donors did contain staphylococcal species, and potentially S. aureus, but also *Corynebacterium* species, which are known to keep S. aureus under control as a commensal rather than a virulent phenotype (45). Although not experimentally proven, this may explain why we did not observe such cytotoxic stress from the nasal microbiota, despite the presumed presence of S. aureus. Scanning electron microscopy images of differentiated immortalized primary nasal epithelial cells cocultured with natural nasal communities for 48 h did not show indications of a negative impact on the cell layer (29), indicating that this type of model system can sustain a natural nasal community without detrimental effects to the cell layer. With respect to epithelial barrier function, endogenous microbiota has also been shown to support tight-junction functionality.

In a Calu-3 cell-based setup, TLR-2 ligands peptidoglycan and the synthetic bacterial lipopeptide Pam3CSK4 were found to increase epithelial barrier functionality after 20 and 24.5 h, respectively, through upregulation of tight junction proteins claudin-1 and zonula occludens 1 (46).

Incubation of Calu-3 cells with nasal swabs for 72 h also allowed monitoring the microbial community. The microbiome of the sinonasal cavities is characterized by the high relative abundance of three phyla: *Firmicutes*, *Actinobacteria*, and *Proteobacteria* in different proportions (47–50). We detected a similar pattern of high relative abundances of these three phyla, and to a lesser extent *Bacteroidetes*, in our model system. De Boeck et al. (51) describe the existence of community types in the nose and nasopharynx. The donor-derived microbiota in our experiment corresponded to the most prevalent community type in their study (91% of all samples), namely, the intermixed *Staphylococcus*, *Dolosigranulum*, *Corynebacterium* type. Interestingly, during the dual coculture experiment, we observed a time-dependent shift from a *Corynebacterium*-dominated to a *Staphylococcus*-dominated community upon transition from the nasal cavity (swab sample) to the *in vitro* epithelial cell layer. Charles et al. (29) cocultured nasal bacteria on immortalized primary epithelial cell layers and found a consistent increase of *Staphylococcus* spp. in cocultured communities compared to the original inoculum, indicating that this *in vitro* environment favored the growth of certain community members, including *Staphylococcus* spp. Staphylococcal species are found in the nasal cavity of most individuals, in particular S. epidermidis and S. aureus (50–55). Both species are equipped to adapt to the nutrient-scarce environment of the nasal cavity (56) and to attach to airway epithelium, are able to evade clearance by the immune system, and can produce antimicrobial peptides and proteases, which allows them to outcompete other bacteria in this niche (reviewed for S. aureus in reference 27). With respect to the *Actinobacteria* phylum, *Corynebacterium* and *Propionibacterium* species are typically in high abundance in most individuals (47, 48, 50, 54, 57). Although *Corynebacterium* was an important colonizer in our model system, OTU belonging to the genus *Propionibacterium* were only scarcely detected. Colonization of P. acnes, recently reclassified as Cutibacterium acnes (58), is favored by the presence of sebum from sebaceous glands (59), and yet these are not present in the mucosal epithelium of the posterior nares and the sinuses. Since our objective was to establish a mucosal epithelial model of the anterior area, we took care to avoid contact with the anterior nose during sampling of the inoculum. Because *Propionibacterium* OTU in the middle meatus and sphenoid recess are typically low compared to the anterior nares (50), a high abundance of *Propionibacterium* sp. was not expected. Finally, from the *Proteobacteria* phylum, the genus *Salmonella* was a profound colonizer of our *in vitro* model. *Salmonella* is less commonly described in the human sinonasal cavities, except for rare cases of reptile-associated maxillary sinusitis (60). It has been suggested that species can (transiently) occupy the respiratory tract through microaspiration from the oral cavity (16, 61), where the presence of *Salmonella* species has been reported.

As indicated by the phenotypic and phylogenetic alpha diversity, our Calu-3/microbiota coculture setup displayed a transition in the microbiome that reached steady state after 48 h. This might indicate a stabilization period during which adaptation and new homeostasis of the bacterial community in the model environment is established. Nevertheless, Chao1 estimators of richness in the coculture setup were similar to those reported from nasal communities in healthy individuals (41, 51, 54). While alpha diversity levels in the model system at the time of inoculation were somewhat lower than those observed *in vivo* (50, 54, 62), we observed a decrease in alpha diversity over time. Such a reduction in phylogenetic diversity upon inoculation of a natural community is indicative of a selective effect under the more restricted growth conditions *in vitro* and has been described before for oral epithelial model systems (63). The diminution in diversity in our Calu-3/nasal microbiota model was attributed to a loss of evenness in the community, largely due to the increase of *Otu0001*, belonging to the genus *Staphylococcus*, and *Otu0002*, belonging to the genus *Salmonella*. To better understand and mimic *in vivo* processes, it is necessary to recreate the *in situ* environment, especially when community assembly is strongly dependent on abiotic and biotic selection (64), as is the case in the (chronically inflamed) sinonasal cavities.

Our model system could benefit from further characterization, and the ability to determine bacterial localization in and on the epithelial structure can be valuable for understanding disease exacerbations and persistence, e.g., in chronic rhinosinusitis. Localization of the bacterial community and/or its subpopulations can be achieved through different approaches. Planktonic bacterial cells or loosely associated cells can be retrieved through washing of the cell layers, as described here. Bacterial cells that are attached to the cell layer or are internalized in the cell layer can be retrieved after washing off the loosely associated bacteria and planktonic bacteria. Differentiation between bacteria attached to the cell layer and internalized bacteria can be accomplished via antibacterial treatment, followed by propidium monoazide treatment and DNA extraction (65). If pure bacterial cultures (or a defined mixture thereof) are studied, antibiotic treatment followed by culturing of the internalized bacteria can be applied (66). In biofilm-mediated diseases, and also as observed in CRS patients, chronic disease, extreme resistance to antibiotic treatment, and repeated acute exacerbations are characteristic of biofilm formation (67, 68). Biofilm formation in our model system can be determined through microscopy or biofilm-staining procedures (69, 70). The distribution of specific (known) subpopulations of the bacterial community in the model system can be assessed using fluorescence *in situ* hybridization, similar to the elegant approach of Welch et al. (71, 72) to study biogeography in host environments.

The second modulatory component of URT disease etiology was introduced by inclusion of THP-1-derived macrophages-like cells at 10^5^ cells per well in our experimental setup. With respect to epithelial integrity, slightly lower TEER values were found compared to cocultures in the absence of macrophages. However, over the 72-h incubation period the TEER values remained more stable. Coculture of Calu-3 cell layers with THP-1-derived macrophage-like cells are known to decrease epithelial resistance when seeded at concentrations of 2 × 10^5^ cells ml^−1^, and yet this effect is not apparent when lower seeding densities (2 × 10^4^ cells ml^−1^) are applied (73). Similar drops in TEER from THP-1-derived macrophages were previously noted for coculture models of the lung-blood barrier (74). While phorbol-12-myristate-12-acetate (PMA), the differentiation agent for THP-1 cells, is known to induce an inflammatory phenotype *in vitro* (75), it is not the cause for lower barrier integrity in our setup since the epithelial cell layers were not exposed to PMA. Whether or not TNF-α secretion by THP-1 cells was responsible for lower TEER values in our setup, as previously demonstrated for Caco-2/THP-1 cell cocultures (76), was not investigated in our study. However, an increased TNF-α release was measured in our triple coculture setup compared to the dual coculture setup, which might indicate a role of this cytokine in the observed decrease in epithelial resistance.

We completed our URT experimental model and created a triple coculture system by inclusion of healthy donor-derived microbiota. From the two donors tested no adverse effects on epithelial barrier function were noted. In an additional experiment, we assessed the effects of S. aureus and *L. sakei* in our triple coculture setup. Similar to the dual coculture setup, coculture with S. aureus resulted in an increase in cytotoxicity and inflammatory cytokine (IL-8 and TNF-α) production and a loss of epithelial integrity, whereas this was not observed in the sterile control or the *L. sakei*-inoculated cell layers. Bacterial growth was similar in both setups, indicating that the inclusion of THP-1-derived macrophages did not affect the growth of these bacterial species. This demonstrates the ability of our triple coculture, now including epithelial cells and immune cells, to maintain a healthy epithelial layer for prolonged exposure to nasal microbiota from *in vivo* human origin. Interestingly, interindividual variability in nasal microbiota background resulted in a variable immune response in our model system for IL-8 and yet not for TNF-α. The introduction of macrophage-like cells caused increasing IL-8 levels in cell layers inoculated with material from donor 1, whereas for donor 2 a mild decrease in IL-8 levels was noted after 72 h. This IL-8 release was not reflected in absolute cytotoxicity values, yet normalized to protein content, incubation with donor 1 derived microbiota did show increased cytotoxicity. However, cytotoxic stress stayed below 10% for all groups and time points; this again underlines the feasibility of our experimental setup to sustain coculture of epithelial and immune cells with a natural community from healthy human origin. Donor-specific differences in cytokine release and cytotoxicity illustrate the modulating effects of a microbial community on cellular physiology and emphasize the importance of incorporating the sinonasal microbial community in *in vitro* model systems.

A highly valuable observation from this study is that presence of THP-1-derived macrophage like cells is a determinant of microbiome composition in the triple coculture experiment. Phenotypic alpha diversity was higher in microbial communities sampled from epithelial cell layers cocultured with macrophage-like cells than cell layers without macrophage-like cells. Moreover, bacterial communities in the triple coculture setup were dominated by *Mycoplasma* sp. and *Moraxella* sp., whereas the dual coculture setup without macrophages primarily displayed abundance of *Corynebacterium* sp., *Staphylococcus* sp., and *Dolosigranulum* sp. Together with the epithelium, macrophages are indispensable in sensing the environment and act as a first line of (cellular) defense against pathogenic insults in the upper respiratory tract. Cross talk between macrophage-like cells and the respiratory epithelial cell layer has been shown to attenuate the response toward pathogen infection in a mechanism that could potentially serve to avoid severe tissue damage by inflammation (35). Next to this, (alternatively activated) macrophage infiltration in (polyp) tissue in chronic rhinosinusitis with nasal polyps could play a role in disease perpetuation (30–32, 34). Through the enforcement of an inflammatory response or the induction of a more immunotolerant environment upon repeated exposure (35, 77, 78), the tissue macrophages can, directly and indirectly through the epithelium, shape the microbial community by preventing the outgrowth of particular bacteria. This relationship is bidirectional, since the resident microbiota and its metabolites will induce (anti-)inflammatory responses in the human host.

Coculturing human respiratory epithelial cells in direct contact with pure cultures of microorganisms—including pathogens—and natural nasal microbiota is feasible provided *in vivo* representative microbial loads are used. As indicated by absence of cytotoxicity and stable cytokine profiles and epithelial integrity, nasal microbiota from human origin appear to be well tolerated by host cells, while microbial community composition remained representative for that of the human (sino)nasal cavity. Importantly, the introduction of macrophage-like cells enabled us to obtain a differential readout from the epithelial cells dependent on the donor microbial background to which the cells were exposed. We conclude that both model systems offer the means to investigate host-microbe interactions in the URT.

## MATERIALS AND METHODS

### Human cell culture.

Calu-3 (ATCC HTB-55, lot 62657853; ATCC, LGC Standards) cells were cultured at 37°C, 10% CO_2_, and 90% relative humidity in MEM (Gibco) supplemented with 10% (vol/vol) heat-inactivated fetal bovine serum (FBSi; Greiner Bio-One, lot 10635093). Cells were used at passages 26 and 27. An antibiotic/antimycotic solution of streptomycin-penicillin-amphotericin B (antibiotic antimycotic solution 100×; Sigma-Aldrich) was added (1% [vol/vol]) to the growth medium for routine culture. Prior to seeding in Transwell inserts, cells were stained with trypan blue (0.4% [wt/vol]) in phosphate-buffered saline [PBS]; Sigma-Aldrich) to assess viability and counted using a Neubauer counting chamber. Transwell inserts (Corning) with polyester (PET) membranes (pore size, 0.4 μm; growth area, 4.67 cm^2^) were seeded with 10^5^ cells cm^−2^ and maintained under submerged conditions until fully confluent. When confluent, the cell layers were brought to the air-liquid interface (ALI) for differentiation for 21 and 43 days, respectively. At 7 days prior to the bacterial inoculation, the medium was switched to FBSi-supplemented medium without antibiotic/antimycotic solution.

### Donor recruitment and sampling.

Nasal swabs were collected from healthy donors. The inclusion criteria were clinical health, no history of smoking, aged between 18 and 65, no asthma or atopy, no antibiotic use in the past 4 weeks, no use of topical corticosteroids in the past 4 weeks, and no history of chronic or recurrent respiratory illnesses such as (chronic) rhinosinusitis, chronic obstructive pulmonary disease, or aspirin-exacerbated respiratory disease. Research incubation work with nasal microbiota from human origin was approved by the ethical committee of the Ghent University hospital under registration number B670201214538. Two nasal swabs (sterile; Novolab, Belgium) were taken from the posterior nose, and care was taken to avoid contamination from the nostrils. One swab was stored at –20°C until further analysis. The other swab tip was placed in an Eppendorf tube in sterile PBS and placed on a Vortex shaker during 60 s. The tip was then removed from the tube, and dilutions were prepared for flow cytometry and inoculation of the respiratory tract cell layers.

### Bacterial culture.

Pure strains of Staphylococcus aureus (LMG 16217) and Lactobacillus sakei (LMG 21529) were retrieved from a –80°C glycerol stock. The pure strains were cultured at 37°C in lysogeny broth (LB) agar (Lennox, Carl Roth) and then transferred to LB (Lennox, Carl Roth) (37°C).

### Coculture experimental setups. (i) Dual coculture of respiratory epithelial cells and nasal microbiota.

**FIG 5:**
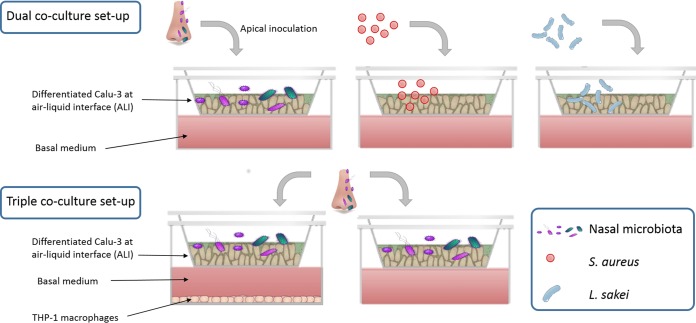
Schematic representation of the dual and triple coculture setups and experiments.

Transwell inserts with differentiated cell layers (transepithelial electrical resistance, ≥500 Ω) were inoculated after 21 days at ALI with either pure cultures of S. aureus (*n* = 5) or *L. sakei* (*n* = 5) or the donor-derived community (1 donor, *n* = 5) from nasal swab samples (Fig. 5). Control cell layers (*n* = 5) were sham inoculated with sterile PBS. Prior to inoculation, bacterial cultures were washed twice with sterile PBS and then suspended in sterile PBS and counted using flow cytometry. After adjustment of the bacterial cell concentration, 200 μl of the suspension was pipetted onto the apical side of the differentiated cell layer (10^3^ intact bacterial cells per insert), after which it was left to incubate for 2 h at 37°C. Subsequently, the inoculation medium was aspirated to restore ALI culture, removing nonadherent microbial cells. The aspirated inoculation medium was then counted using flow cytometry to calculate the proportion of bacterial cells remaining on the cell layers (remaining intact bacterial cells = inoculated intact bacterial cells – aspirated intact bacterial cells). Cocultures were incubated at 37°C, 10% CO_2_, and 90% relative humidity for 3 days. During the coculture of epithelial cells with bacterial cells, the apical side of the differentiated cell layer was washed daily with 1 ml of sterile PBS at 37°C.

### (ii) Triple coculture of respiratory epithelial cells, immune cells, and nasal microbiota.

In this assay, a comparison is made between dual coculture (Calu-3 plus microbiota) and triple coculture (Calu-3 plus THP-1 plus microbiota) setups inoculated with nasal bacteria from identical donors (Fig. 5). Calu-3 cells were seeded on the apical side of Transwell inserts in 6-well plates and maintained until fully confluent as described in the previous section. Cells were cultured at ALI for 43 days, until TEER values of ≥500 Ω for each insert were obtained. At 1 week before the assay, the cell culture medium was switched to medium without antibiotics and without fungicides to allow coculture with microorganisms. THP-1 (ATCC TIB-202, passages 43 to 50) cells were seeded in 6-well plates at a density of 10^5^ cells/well in RPMI 1640 medium with GlutaMAX supplemented with 10% FBSi. Monocytes were differentiated into macrophages using phorbol-12-myristate-12-acetate (PMA; Cayman Chemicals) at a concentration of 20 ng ml^−1^ for 24 h. THP-1-derived macrophages and Calu-3 cell layers were combined prior to bacterial inoculation by placing the inserts with differentiated Calu-3 cell layers in the 6-well plate containing THP-1-derived macrophages. Nasal microbiota samples were obtained from two healthy donors, different from the previously described assay, and for each donor we inoculated Calu-3 cell layers in coculture with THP-1 cells (*n* = 3) and Calu-3 cell layers without THP-1 cells (*n* = 3).

### Sham-inoculated cell layers (*n* = 3) were included for setups with or without THP-1 cells.

The same inoculation procedure used for the previous experiment was used with 10^4^ intact bacterial cells per insert. To examine the viability of the triple coculture setup with donor communities, we included treatment groups that were either inoculated with 10^4^ intact cells of L. sakei (commensal/probiotic) or S. aureus (pathogen/pathobiont) to compare the effects on cytokine production, cytotoxicity, and epithelial resistance of the respiratory cell layers.

### Cytokine secretion.

Secretion of cytokines IL-8, TNF-α, and IL-10 into the basolateral compartment by the mammalian cells was measured with an enzyme-linked immunosorbent assay (ELISA) prior to inoculation and at *t* = 0, 2, 24, 48, and 72 h after inoculation. IL-8 was measured with a human IL-8 (CXCL8) Mini-TMB ELISA development kit (PeproTech) for the dual coculture assay, with a human IL-8 (CXCL8) standard ABTS ELISA development kit (PeproTech) for the triple coculture assay, and with a human IL-8 uncoated ELISA kit (Invitrogen) for the additional triple coculture assay with *L. sakei* and S. aureus, all performed according to the manufacturer’s instructions. IL-10 and TNF-α were measured with a human IL-10 Mini-ABTS ELISA development kit (PeproTech) according to the manufacturer’s instructions. Color development was measured with an Infinite M200 Pro plate reader (Tecan) at 405 nm with wavelength correction at 650 nm for ABTS substrate and at 450 nm with wavelength correction at 620 nm for TMB substrate.

### Cytotoxicity.

Cytotoxicity of the epithelial layers was assessed with the lactate dehydrogenase (LDH) assay. LDH that leaked from the cell layer in the basolateral compartment was measured using a Pierce LDH cytotoxicity assay kit (Thermo Fisher Scientific) according to the manufacturer’s instructions. Samples of the medium were taken from the basolateral compartment prior to inoculation and at *t* = 0, 2, 24, 48, and 72 h after inoculation. Absorbance at 490 nm with wavelength correction at 680 nm was measured with an Infinite M200 Pro plate reader (Tecan). Spontaneous LDH release was calculated as the mean LDH release from three representative differentiated cell layers at *t* = 0 h, prior to inoculation (dual coculture) or from designated, nontreated cell layers with or without macrophage-like cells (*n* = 3). Maximal LDH release was calculated as the mean LDH release from three lysed cell layers (dual coculture) or three lysed cell layers with or without THP-1-derived macrophages (triple coculture).

### Protein content.

Cell layers were washed twice with sterile PBS, scraped off the Transwell inserts using sterile cell scrapers (20 mm wide; Bedalab), suspended in 1 ml of Hanks balanced salt solution (Gibco), and centrifuged at 125 × *g* for 7 min. The supernatant was removed, and the samples were stored at –20°C until further processing. The total protein content of the cell layers was determined using a Bradford assay (Bio-Rad) according to the manufacturer’s instructions.

### Transepithelial electrical resistance.

The TEER of the cell layers was assessed during differentiation using an epithelial volt-ohmmeter (MilliCell ERS-2; Millipore) with an MERSSTX01 electrode. TEER measurements were performed twice per insert to account for electrode variability. The measured TEER values were corrected by subtracting the TEER value of a Transwell insert without cells under identical conditions and are given in ohms. To allow comparison of the treatments between and within groups during the triple coculture experiment, TEER values of each cell layer were scaled to the TEER value at the start of the experiment and are therefore presented as percentages. TEER values were measured similarly during the coculture experiment, but with 2.5 ml of cell culture medium in the basolateral compartment and 1 ml of PBS in the apical compartment. The chopstick electrode was sterilized with 70 vol% ethanol in water and rinsed with cell culture medium after each insert measurement. During the coculture experiments, TEER values were obtained at *t* = 0, 24, 48, and 72 h.

### Intact/damaged staining and flow cytometry.

Bacterial cell densities were measured using an AccuriC6 flow cytometer and intact cells were distinguished from damaged cells using SYBR Green (SG; Thermo Fisher)/propidium iodide (PI; Sigma) staining, as previously described (79). During coculture, bacterial cells were recovered from the cell layer by gently pipetting up and down the apical washing fluid (PBS with calcium and magnesium ions), and the bacterial densities in the apical washing fluid were determined by using flow cytometry.

### Flow cytometric fingerprinting.

Flow cytometric fingerprints were calculated using the R package Phenoflow (80). Fingerprints were constructed based on forward scatter, sideward scatter, SG fluorescence, and PI fluorescence of the sample using 128 bins per channel (nbin = 128) and a bandwidth of 0.01 (bw = 0.01) for the kernel density estimator calculated at each bin. Fingerprint-based ecological parameters (i.e., alpha and beta diversities) were calculated using the *Diversity_rf*() function and the *beta_div_fcm*() function from the R package Phenoflow (80). Phenotypic beta diversity was calculated using Bray-Curtis distances. NMDS was used to create ordination plots of the beta diversity.

### DNA extraction.

DNA was extracted from swab samples and from 500 μl apical washing fluid using the MO BIO PowerSoil DNA isolation kit (MO BIO) according to the manufacturer’s protocol (dual coculture experiment). Samples were subsequently diluted ten times in Tris-EDTA (TE) buffer (VWR) to further avoid PCR inhibition. DNA from samples from the triple coculture experiment was extracted by using a physicochemical lysis protocol, followed by phenol extraction (see Text S1 in the supplemental material), as previously described (81).

10.1128/mSphere.00916-19.7 TEXT S1 DNA extraction. Download Text S1, RTF file, 0.1 MB. Copyright © 2020 De Rudder et al. 2020 De Rudder et al. This content is distributed under the terms of the Creative Commons Attribution 4.0 International license.

### 16S rRNA gene sequencing and data analysis.

The PCRs included about 1 to 10 ng of DNA extract (total volume, 1 μl), 15 pmol of each forward primer and reverse primer (in 20 μl of 1× MyTaq buffer containing 1.5 U of MyTaq DNA polymerase; Bioline) and 2 μl of BioStabII PCR enhancer (Sigma). For each sample, the forward and reverse primers had the same 10-nucleotide barcode sequence. PCRs were carried out for 30 cycles using the following parameters: 96°C for 2 min (predenaturation), followed by 96°C for 15 s, 50°C for 30 s, and 70°C for 90 s. The DNA concentration of amplicons of interest was determined by gel electrophoresis. Approximately 20 ng of amplicon DNA from each sample was pooled for up to 48 samples carrying different barcodes. The amplicon pools were purified with one volume AMPure XP beads (Agencourt) to remove primer dimer and other small mispriming products, followed by an additional purification on MinElute columns (Qiagen). About 100 ng of each purified amplicon pool DNA was used to construct Illumina libraries using the Ovation Rapid DR Multiplex System 1-96 (NuGEN). Illumina libraries were pooled and size selected by preparative gel electrophoresis. Sequencing was done on an Illumina MiSeq using V3 chemistry (Illumina) by LGC Genomics.

The amplicon data were processed as described in De Paepe et al. (82) using mothur (v1.39.5), largely based on the protocol by Kozich et al. (83). First, forward and reverse reads were assembled into contigs. Contigs with ambiguous base calls or nonsufficient overlap were removed. The remaining contigs were aligned to the mothur-formatted SILVA SEED (release 128) alignment database, trimmed between positions 2 and 17012 to be compatible with the 341F (forward) and 785Rmod (reverse) primer set (90). Contigs not aligning with this region or containing homopolymer stretches of more than 12 bases were removed. A preclustering step was then performed, clustering sequences with a maximum of four differences (1% difference). A chimera check was performed using UCHIME (84), and chimeric sequences were removed. The sequences were then classified using the RDP 16S rRNA gene training set (v14) by means of a naive Bayesian classifier (Wang’s algorithm [85]). Sequences classified as eukaryota, chloroplasts, and mitochondria were removed. Sequences not classifiable at the (super)kingdom level were removed as well. The OTU (operational taxonomic units) were clustered with an average linkage and at the 97% sequence identity. OTU with only a single read were removed. Unique and total sequences retained after every step are presented in Tables S1 and S2 in the supplemental material. Due to the experimental setup, samples from the dual coculture assay at *t* = 24 h and *t* = 0 h had low bacterial cell densities, and less DNA could be extracted from these samples. These samples yielded <1,000 reads after sequence data processing. We included these samples for illustrative purposes, and yet we note that they should be interpreted cautiously. During the triple coculture assay, only samples from *t* = 72 h were sequenced for the same reason. Alpha diversity measures were calculated on samples that were sequenced sufficiently deep, as visually assessed by verifying whether the rarefaction curves levelled off (86). Collector curves were constructed for alpha diversity estimators, and only stable estimators (collector curves independent of sequencing depth) were used for further analysis. Richness was estimated using the Chao1 index (39), and diversity was estimated by using the Shannon index and the inverse Simpson index. NMDS plots were constructed using Bray-Curtis distances (stress = 0.0377 for dual coculture experiment, and stress = 0.063 for triple coculture experiment), and the significance of clustering observed in these plots was assessed using permutation multivariate analysis of variance (PERMANOVA [87]) using the adonis function (R package vegan [86]) based on the Bray-Curtis distance. Significant differences in OTU abundance between time points were assessed using differential expression analysis by means of the DESeq2 package (40). Representative sequences for OTU with significantly different abundances between time points classified using the NCBI BLAST tool (16S rRNA gene sequences, highly similar sequences, megablast [accessed July 2018]), the RDP SeqMatch online tool (accessed July 2018), and the HOMD 16S rRNA sequence identification tool (expanded Human Oral Microbiome Database, eHOMD [accessed July 2018]).

10.1128/mSphere.00916-19.5 TABLE S1 Unique and total sequences during dual coculture sequence processing steps. Download Table S1, DOCX file, 0.01 MB. Copyright © 2020 De Rudder et al. 2020 De Rudder et al. This content is distributed under the terms of the Creative Commons Attribution 4.0 International license.

10.1128/mSphere.00916-19.6 TABLE S2 Unique and total sequences during triple coculture sequence processing steps. Download Table S2, DOCX file, 0.01 MB. Copyright © 2020 De Rudder et al. 2020 De Rudder et al. This content is distributed under the terms of the Creative Commons Attribution 4.0 International license.

### Statistical analysis.

All statistical analyses were performed using R version 3.4.2 (The R Foundation for Statistical Computing) and the following packages: vegan (86), phyloseq (88), DESeq2 (40), Phenoflow (80), and PMCMR (89). The packages ggplot2 and viridis were used for plotting. The normality of the data was assessed using the Shapiro-Wilk test, and homoscedasticity was assessed with the Bartlett test for equal variances. In case normality and homoscedasticity were rejected, the Kruskal-Wallis test (K-W) was used for multiple comparisons, followed by Dunn’s *post hoc* test with Holm’s correction. When normality and homoscedasticity were not rejected, two-way ANOVA was performed, followed by Tukey’s HSD *post hoc* test. Significance was considered at the 5% level (α = 0.05).

### Data availability.

Sequences of swabs and cocultured communities are available at the NCBI Sequence Read Archive under BioProject accession number PRJNA550035.
