# Supplementary figures and images for: Dual and Triple Epithelial Coculture Model Systems with Donor-Derived Microbiota and THP-1 Macrophages To Mimic Host-Microbe Interactions in the Human Sinonasal Cavities
Source: mSphere. 2020 Jan 15;5(1):e00916-19. doi: 10.1128/mSphere.00916-19 (PMC6968656; doi:10.1128/mSphere.00916-19)

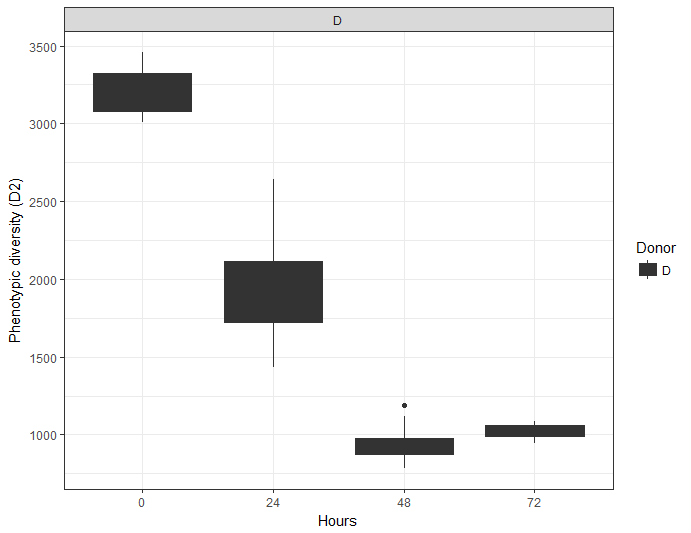

Supplement: FIG S1 [file mSphere.00916-19-sf001.tif]

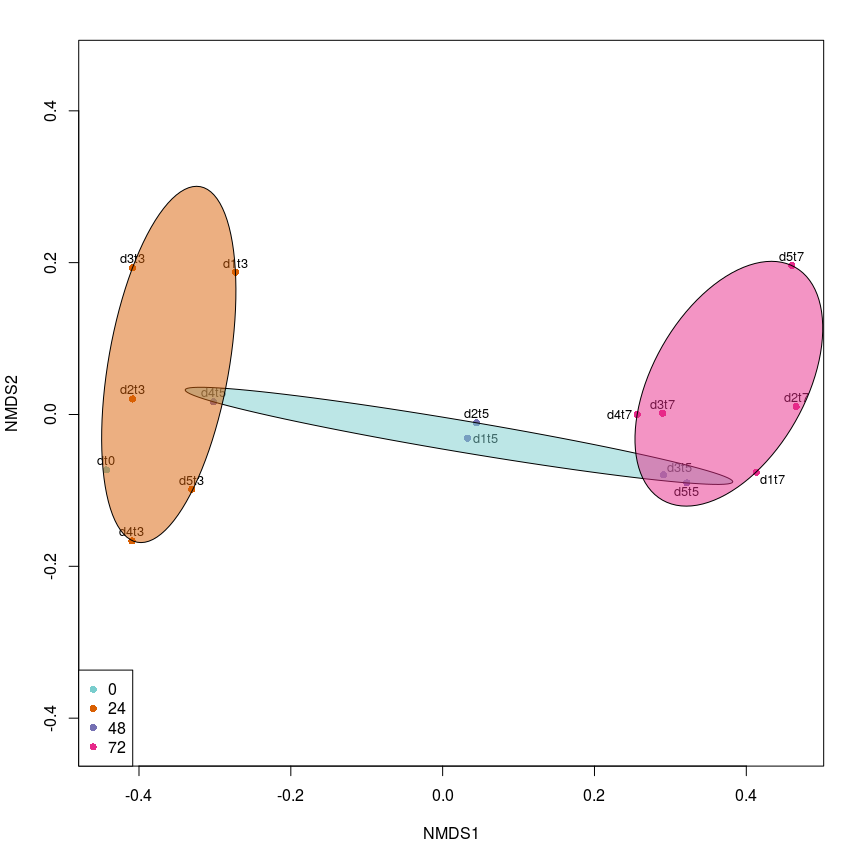

Supplement: FIG S2 [file mSphere.00916-19-sf002.tif]

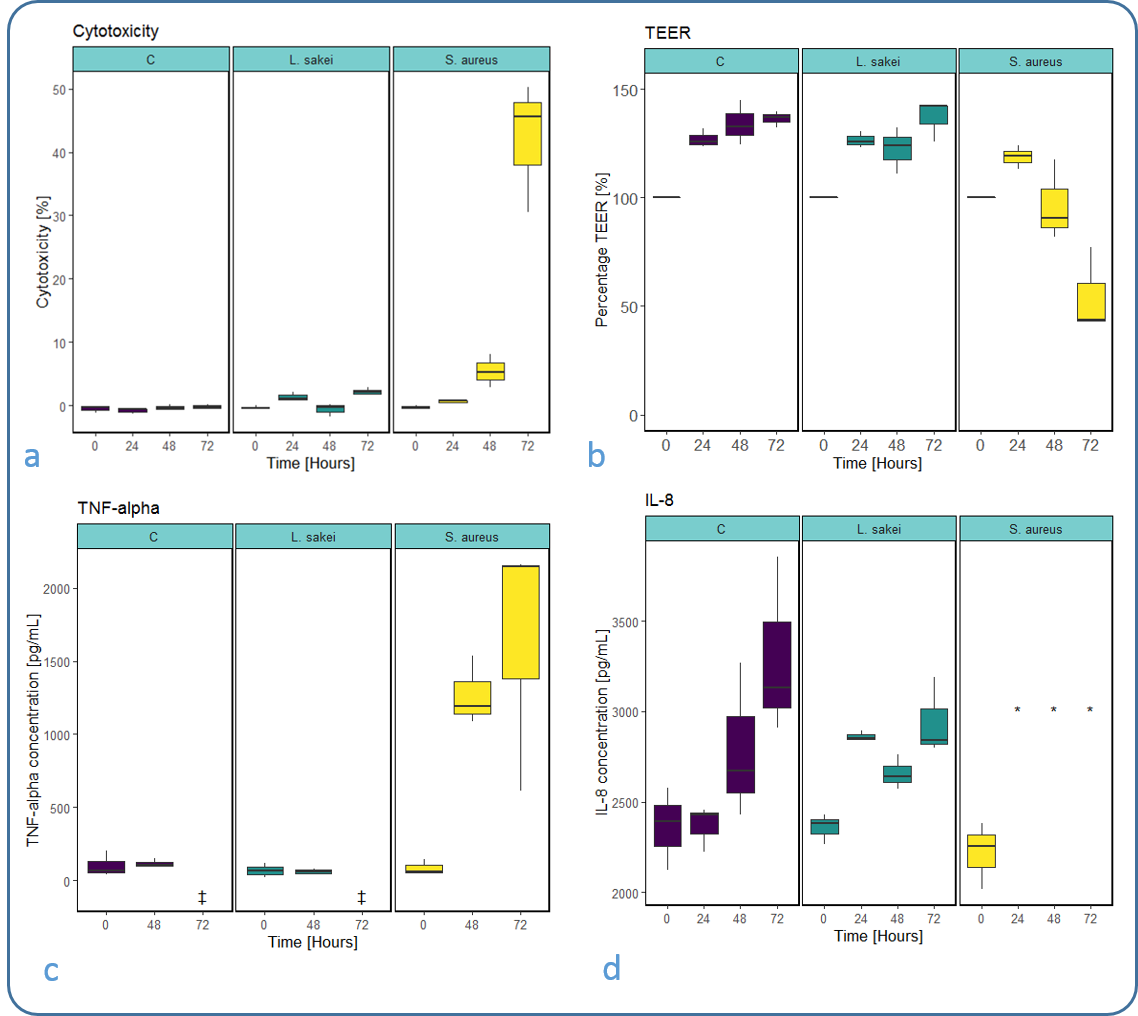

Supplement: FIG S3 [file mSphere.00916-19-sf003.tif]

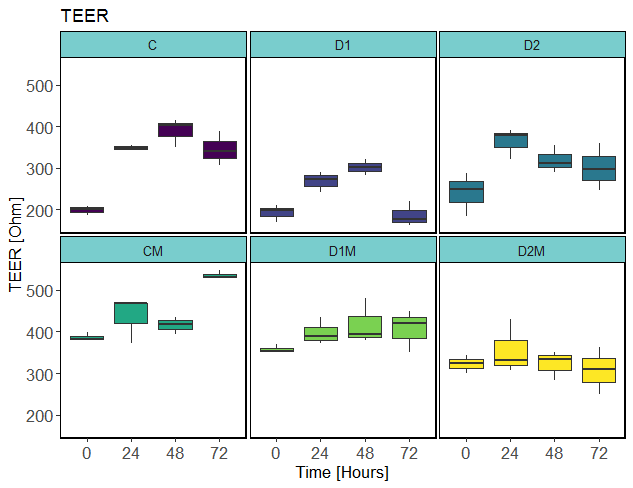

Supplement: FIG S4 [file mSphere.00916-19-sf004.tif]
