# Supplementary material for: Dual and Triple Epithelial Coculture Model Systems with Donor-Derived Microbiota and THP-1 Macrophages To Mimic Host-Microbe Interactions in the Human Sinonasal Cavities
Source: mSphere. 2020 Jan 15;5(1):e00916-19. doi: 10.1128/mSphere.00916-19 (PMC6968656; doi:10.1128/mSphere.00916-19)
Supplement: TEXT S1 [file mSphere.00916-19-s0001.rtf]

1  DNA Extraction
In short, samples were mixed with lysis buffer, containing 100 mM Tris pH 8, 100 mM EDTA pH 8, 100 mM NaCl, 1 % polyvinylpyrrolidone (PVP40) and 2 % sodium dodecyl sulphate (SDS). 200 mg of 0.1 mm glass beads was added to the samples after which they were disrupted in the PowerLyzer (MoBio) at 2000 rpm for 300 seconds. The samples were centrifuged at maximum speed for 5 minutes and the supernatant was added to a new tube containing 500 ìL of phenol:chloroform:isoamilic alcohol 25:24:1 at pH 7. After mixing and subsequent centrifugation, the upper phase was added to a new tube containing 700 ìL of chloroform. After mixing and centrifugation, 450 ìl of the upper phase was added to a new tube containing 500 ìL of cold isopropanol and 45 ìL of 3M sodium acetate. The samples were mixed and stored at -20 °C for one hour after which they were centrifuged at 4 °C for 30 minutes. The supernatant was removed and the DNA pellet was dried prior to dissolving in 1X TE [Vilchez-Vargas et al., 2013].  
References
R. Vilchez-Vargas, R. Geffers, M. Suárez-Diez, I. Conte, A. Waliczek, V. S. Kaser, M. Kralova, H. Junca, and D. H. Pieper. Analysis of the microbial gene landscape and transcriptome for aromatic pollutants and alkane degradation using a novel internally calibrated microarray system. Environmental Microbiology, 15 (4): 1016–1039, 2013. 
