# Supplementary material for: Dual and Triple Epithelial Coculture Model Systems with Donor-Derived Microbiota and THP-1 Macrophages To Mimic Host-Microbe Interactions in the Human Sinonasal Cavities
Source: mSphere. 2020 Jan 15;5(1):e00916-19. doi: 10.1128/mSphere.00916-19 (PMC6968656; doi:10.1128/mSphere.00916-19)
Supplement: TABLE S1 [file mSphere.00916-19-st001.docx]

| Step | Total |  | Unique |
| --- | --- | --- | --- |

Contigs 78934 -

| Initial trim | 42372 |  | 13254 |
| --- | --- | --- | --- |
| Precluster | 42306 |  | 2437 |
| UChime | 41871 |  | 2150 |
